# A Tridimensional Model for NK Cell-Mediated ADCC of Follicular Lymphoma

**DOI:** 10.3389/fimmu.2019.01943

**Published:** 2019-08-14

**Authors:** Emilie Decaup, Cédric Rossi, Pauline Gravelle, Camille Laurent, Julie Bordenave, Marie Tosolini, Anne Tourette, Emeline Perrial, Charles Dumontet, Mary Poupot, Christian Klein, Ariel Savina, Jean-Jacques Fournié, Christine Bezombes

**Affiliations:** ^1^Centre de Recherches en Cancérologie de Toulouse (CRCT), UMR1037 INSERM, Université Toulouse III: Paul-Sabatier, ERL5294 CNRS, Université de Toulouse, Toulouse, France; ^2^Laboratoire d'Excellence TOUCAN, Toulouse, France; ^3^Programme Hospitalo-Universitaire en Cancérologie CAPTOR, Toulouse, France; ^4^CHU Dijon, Hématologie Clinique, Hôpital François Mitterand, Dijon, France; ^5^Department of Pathology, Institut Universitaire du Cancer de Toulouse, Toulouse, France; ^6^Pôle Technologique du Centre de Recherches en Cancérologie de Toulouse, Toulouse, France; ^7^INSERM1052/CNRS5286/Université Claude Bernard, Lyon, France; ^8^Roche Pharmaceutical Research and Early Development, Roche Innovation Center Zurich, Schlieren, Switzerland; ^9^Institut Roche, Boulogne-Billancourt, France

**Keywords:** ADCC—antibody dependent cellular cytotoxicity, NK cells, modelization, 3D co-culture model, follicular lymphoma

## Abstract

Follicular lymphoma (FL) is the second most frequent subtype of B non-Hodgkin's lymphomas (NHL) for which the treatment is based on the use of anti-CD20 mAbs. NK cells play a crucial role in their mechanism of action and the number of these cells mediating antibody-dependent cell cycotoxicity (ADCC) in the peripheral blood of FL patients predict the outcome. However, their presence in FL biopsies, their activation and their role have been poorly investigated. Moreover, *in vitro* studies have not deciphered the exact signaling cascades triggered by NK cells in presence of anti-CD20 mAbs on both effector and target cells in a relevant FL model. We performed *in silico* analyses and *ex vivo* functional assays to determine the presence and the activation status of NK cells in FL biopsies. We modelized ADCC phenomenon by developing a co-culture model composed by 3D-cultured FL cells and NK cells. Thus, we investigated the biological effect of anti-CD20 mAbs by fluorescent microscopy and the phosphorylation status of survival pathways by cell bar coding phosphoflow in target cells. In parallel, we measured the status of activation of downstream FcγRIIIa signaling pathways in effector cells and their activation (CD69, perforin, granzyme B, IFNγ) by flow cytometry. We determined by *in vivo* experiments the effects of anti-CD20 mAbs in presence of NK cells in SCID-Beige engrafted FL mice. Here, we show that functional NK cells infiltrate FL biopsies, and that their presence tends to correlate with the survival of FL patients. Using our 3D co-culture model, we show that rituximab and GA101 are able to promote degranulation, CD69 expression, IFNγ production and activate FcγRIIIa signaling cascade in NK cells, and inhibit survival pathways and induce apoptosis in FL cells. The effect of GA101 seems to be more pronounced as observed *in vivo* in a xenograft FL model. This study strongly supports the role of NK cells in FL and highlights the application of the 3D co-culture model for *in vitro* validation.

## Background

Follicular lymphoma (FL) is the second most frequent subtype of B non-Hodgkin's lymphomas (NHL) with 20 percent of all NHLs (1, 2). It is an indolent lymphoma with low progression, an overall survival of 10 years and a median age at diagnosis of 60 years (3). FL cells are counterparts of normal germinal center B-cells, and 90% of FL are characterized by overexpression of Bcl-2, an anti-apoptotic protein, caused by the characteristic translocation *t*_(14, 18)_. FL remains an incurable disease despite numerous therapeutic strategies. Therapies include monoclonal antibodies (mAbs) such as the type I anti-CD20 antibody rituximab (RTX), idiotype vaccines, immunomodulatory agents and novel drugs such as kinase inhibitors or bcl-2 inhibitors (4). The combination of chemotherapy and RTX was established as standard care of treatment for FL. Therapeutic mAbs deplete tumor cells through direct or indirect mechanisms of action (5, 6). CD20 engagement induced by Fab binding leads to inhibition of survival pathways and/or direct cell death. By Fc binding *via* complement protein or Fc receptor expressed on cytotoxic cells (NK or γδT lymphocytes) or phagocytic cells (macrophages), anti-CD20 mAbs can induce target cell death through complement dependent cytotoxicity (CDC), antibody dependent cell cytotoxicity (ADCC) or antibody dependent cell phagocytosis (ADCP), respectively. ADCC is considered to be one of the most important mechanisms of action of RTX *in vivo* in mouse models (7) but also in humans as Fc receptor polymorphism is correlated with patient outcome (8).

In order to overcome such resistance, new anti-CD20 mAbs have been developed to improve direct cell death and ADCC (9). Obinutuzumab (GA101) is a humanized glyco-engineered type II anti-CD20 IgG1 mAb. Studies with GA101 in combination with chemotherapy show 93–98% response rates in relapsed and refractory FL patients (10). Based on the data of the Gallium trial where GA101-chemo was directly compared with RTX-chemo, obinutuzumab was approved for 1st line treatment of NHL (11), and based on the Gadolin trial for the treatment of RTX/RTX NHL in combination with bendamustine (12, 13). Unlike type I antibodies, type II antibodies do not translocate CD20 into raft microdomains, but lead to its homotypic aggregation inducing a direct non-apoptotic cell death involving actin rearrangement, lysosomal cathepsin release and generation of reactive oxygen species (14). We also showed a superior direct efficacy of GA101 compared to RTX in 3D FL model called multicellular aggregates of lymphoma cells (MALC) (15). Furthermore, GA101 can induce superior ADCC and ADCP by enhanced binding to FcγRIIIa expressing NK (9, 16, 17), monocytes/macrophages (17) and γδ T cells (18, 19).

NK cells are essential effectors of anticancer immunity and play a crucial role in ADCC (20). Their low count in the peripheral blood of FL patients is correlated with poor prognosis, strongly supporting their key role for therapy (21, 22). In infiltrated lymph nodes and follicles of FL patients, NK cells are present (23), but their cytotoxic status and the modelization of their function in a relevant FL model are unexplored. We are pioneer in the development of 3D FL *in vitro* model, called MALC. MALC recapitulates spatial architecture, gene and protein profiles, and response to drug treatment, making it more relevant than long-used cell suspensions cultures. Such model is powerful to decipher the direct or indirect mechanisms of action of mAbs once cultured in absence (15, 24, 25) or presence of immune cells (26). Moreover, it allows the evaluation of drug penetration and visualization of immune cells infiltration into FL masses as recently published by our group (19). Thus, to investigate NK function in a pertinent FL model, we performed *in vitro* experiments with co-cultures containing NK cells and MALC. We show that anti-CD20 mAbs are able to activate NK by inducing downstream FcγRIIIa signaling cascade and, consequently, mediate target cell death in a 3D model. Moreover, we demonstrate that MALC is a pertinent model for exploring ADCC induced by therapeutic antibodies and models NK cells infiltration in FL biopsies.

## Materials and Methods

### Cells

#### Cell Lines

RL cells were obtained from the American Type Culture Collection (ATCC, Rockville, MD, USA). These cells were cultured in complete RPMI1640 medium at 37°C in humidified 5% CO_2_ atmosphere.

RL-GFP cells were produced by the Inserm UMR1037 vector facility (CRCT, Toulouse, France). Puromycin treatment (0.2 μg/ml) was performed for cell selection.

#### NK Cells

NK cells were isolated from healthy donors. Blood samples were obtained from EFS (Etablissement Français du Sang, Toulouse, France). PBMCs were collected after separation by Ficoll gradient centrifugation. NK cells were isolated from PBMCs using an EasySep Human NK Cell Enrichment Kit (STEMCELL Technologies, Grenoble, France) according manufacter's instructions. NK cells were used when their purity, assessed by flow cytometry, exceeded more than 95%.

#### NK-92 Cells

NK-92 cells used for *in vivo* experiments were obtained by retroviral transduction as described previously (27).

#### Primary FL Cell Isolation and Culture

Lymph nodes were obtained from 19 patients at the Department of Hematology (IUCT, Toulouse, France), who were diagnosed between 2010 and 2014 with FL (Grade I to III-a according to the WHO classification), and who had not received prior therapy (26). The healthy specimens (reactive lymph node, *n* = 7 or normal tonsil, *n* = 10, all from CHU de Toulouse, France) were used as controls. Tissue samples were collected and processed following the standard ethical procedures of the Helsinki protocol, after obtaining written, informed consent from each donor and local ethical committee approval for the study (Comité de Protection des Personnes Sud-Ouest et Outremer II). Cell suspensions from lymph nodes were obtained by dissociating fresh tissues either mechanically either by the use of the gentleMACS™ Octo Dissociator (program m_spleen_04_01). Then, cells were washed in RPMI free medium and freezed at 10.10^6^ cells/vial in human albumin 4%/DMSO 10%, until the anatomopathological diagnosis was performed. For experiments, cells were rapidly defrosted in warm complete medium, filtered through 70 μm nylon cell strainers, and plated at 10.10^6^/mL in complete medium, until the experiment was performed.

### Immunohistochemistry

Three-mm-thick sections of formalin-fixed and paraffin-embedded (FFPE) FL lymph node tissues were stained with an anti-NKP46 antibody (kindly gift by Pr Eric Vivier, Innate Pharma, Marseille, France). Whole slides were digitalized using Pannoramic 250 Flash II digital microscopes (3DHISTECH, Hungary) and read by a pathologist at the IUCT (Toulouse, France). IHC staining was evaluated and percent of NK cells was quantified by automated method using Tissue studio image analysis software (Definiens, Munich, Germany).

### mAbs

Trastuzumab (TTZ, Herceptin), rituximab (RTX, Mabthera) and Obinutuzumab (GA101) were provided by Genentech (San Francisco, USA), Roche (Boulogne-Billancourt, France) and Roche (Zurich, Switzerland), respectively.

### MALC Preparation

MALC were obtained by the hanging drop method described previously (15, 24, 25). Briefly, drops (20 μL) of 10^4^ RL or RL-GFP cells in complete medium enriched with 1% methylcellulose (MethoCult H4230, STEMCELL Technologies, Grenoble, France) were placed onto the lid of a 24-well plate, which was then inverted over a plate containing 1 mL of medium. Hanging drop cultures were incubated for 24 h at 37°C in 5% CO_2_. In parallel, a layer of 1% agarose (Life Technologies, St. Aubin, France) in classical medium was added to another 24-well plate and stored for 24 h at 4°C. After 24 h, the resulting cellular aggregates were transferred to the agarose plate and cultured at 37°C in 5% CO_2_. MALC observation was realized under a microscope Nikon Eclipse TE200 (Nikon, Champigny sur Marne, France) at magnification ×40.

### Detection of MALC Cell Death by Confocal Microscopy

MALC, realized with RL-GFP and obtained after 2 days of growth, were co-cultured with NK cells at ratio 10:1 and treated or not with mAbs at 10 μl/ml. After 4 h, 10 μL of Annexin-V –VPD450 diluted in half in adapted buffer (BD Biosciences) were added on co-cultures. After 1 h of staining, cells were fixed with 4% paraformaldehyde (PFA) overnight at room temperature. Samples were mounted in DABCO mounting medium (Sigma Aldrich, St. Quentin Fallavier, France) into silicone isolator (Invitrogen) and were examined using Zeiss LSM710 confocal microscope (Carl Zeiss, Marly Le Roi, France).

### Flow Cytometry

#### ADCC Assay

MALC obtained after 10 days of growth, were co-cultured with NK cells in a 96-well plate at different effector:target (E:T) ratios (0.5:1 and 1:1) at 37°C and 5% CO_2_ in the presence of anti-CD20 mAbs at 10 μl/mL. PE-Cy5-labeled anti-CD107a or isotype controls (BD Biosciences) were added during the co-culture to assess NK cells degranulation. After 4 h of incubation, co-cultures were mechanically dissociated and labeling solution was added for 15 min at 4°C in the dark. The labeling solution was composed of CD56-PE (Miltenyi Biotec, Bergisch Gladbach, Germany), CD3-PE/Cyanine 7 (clone UCTH1, Clinisciences, Nanterre, France) and CD19-PE (IoTest^®^, Beckman Coulter, Marseille, France). Cells were then washed, stained with DAPI (Sigma Aldrich) and analyzed on a LSRII cytometer (BD biosciences). NK cell degranulation was determined as rate of CD107^+^ cells among the CD56^+^/CD3^−^ population, and FL cell death was determined as rate of DAPI^+^ cells among the CD19^+^ population.

#### Detection of CD69, CD107, Perforin, and IFNγ

MALC obtained at day 10 of growth, were co-cultured with NK cells at ratio E:T 0.5:1 or 1:1 and treated or not with mAbs at 10 μl/ml during 4 h in presence of brefeldin A at 10 μl/mL (except of CD69 detection). Cells were then washed, fixed with 2% PFA during 4 min, washed and permeabilized with 1% saponin during 8 min. Cells were stained with anti-CD56-PE (Miltenyi Biotec), anti-CD3-PE/Cy7 (Beckman Coulter, Roissy, France), anti-CD69-PE-Cy5 (BD Biosciences), anti-perforin (PFN)-FITC (BD Biosciences), anti-IFN-γ-Alexa fluor 647 (BD Biosciences), anti-granzyme B-Alexa647 (BD Biosciences) antibodies during 30 min at 4°C in the dark. Cells were analyzed by flow cytometry with a LSRII flow cytometer (BD Biosciences).

#### Phosphoflow Determination

Phosphoflow was performed as previously described (15, 28). Briefly, MALC obtained at day 10 of growth, were co-cultured with NK cells at ratios E:T 0.5:1 or 1:1 and treated or not with mAbs at 10 μl/ml. After 5, 30, 60, and 240 min, co-cultures were fixed with 2% PFA for 4 min at room temperature, washed and permeabilized with 1% saponin for 8 min at room temperature. Cells were washed and resuspended in cold 50% saponin 1% FCS and mixed. Cell barcoding was then performed by incubating 30 min at 4°C cell barcoding dyes (CBD) 450 and 500 (BD Biosciences) at various concentrations (prepared according to the manufacter's instructions) or dimethyl sulfoxide. Cells were then washed and resuspended in residual volume. Encoded samples were split equally into FACS (Fluorescence-activated cell sorter) tubes for parallel stainings. Conjugated phosphospecific and surface antibodies (against CD3 and CD56) were added to each tube of cells for 30 min at room temperature. Antibodies against P-Akt (S473)-AlexaFluor647, P-Akt (T308)-PE, P-Erk1/2 (T202/Y204), P-p38MAPK (T180/Y182), P-PLCγ2 (Y759), and P-ZAP70 (Y319)/Syk (Y352) were all from BD Biosciences.

### FL Xenograft and ADCC *in vivo*

5 × 10^6^ RL cells were subcutaneously injected in SCID CB 17 mice (females, aged 4 weeks), according to the INSERM Animal Care of Université de Claude Bernard Lyon 1 (CEEA-55) and User Committee-approved protocol (N° DR2015-60). Tumors were measured twice a week using a caliper and tumor volume (TV) was calculated using the formula TV = 4/3 π × r^3^ (r = radius). Once TV reached 100–200 mm^3^, mice were divided in 6 groups. RTX and GA101 were administered by intraperitoneal injection once a week at 30 mg/kg and NK 92 cells (5 × 10^6^ cells) was injected intravenously (in the tail vein) three times a week. Mice were sacrificed when TV reached 1,600 mm^3^.

### Gene Expression Analysis

The GEO Datasets (29) were sourced for FL gene expression profiles obtained with the Affymetrix HG U133 plus 2.0 microarray platform. We downloaded raw data from studies GSE53820 (30), GSE55267 (31), GSE65135 (32), GSE16024 (unpublished), GSE21554 (33), and GSE38816 (34) and normalized these together by the method RMA. Then, these transcriptomes were collapsed to around 21000 HUGO protein-encoding gene symbols. The sample enrichment scores (SES) were computed by Auto-Compare-SES (https://sites.google.com/site/fredsoftwares/products/autocompare_ses) with normalized settings (35). NK and B cell SES signatures were from CIBERSORT (33, 36) based on the expression of genes listed in Supplementary Figure 1. The cytolytic T cell gene signature was defined based on the expression of 8 genes: GZMA, PRFN, GZMB, GZMK, GZMM, GZMH, TRAIL, and IFNG. Whenever specified, Pearson correlations were computed between gene ranks and SES.

### Statistics

Data shown are means ± SD. For comparison of two series of normally distributed variables, we used paired and one-tailed Student's *t*-tests with *p* < 0.05 for statistical significance. When comparing three or more parameters, a one-way ANOVA with Dunnett's multiple comparison *post-test* correction was used.

## Results

### Functional NK Cells Infiltrate FL Lymph Nodes

**Figure 1:**
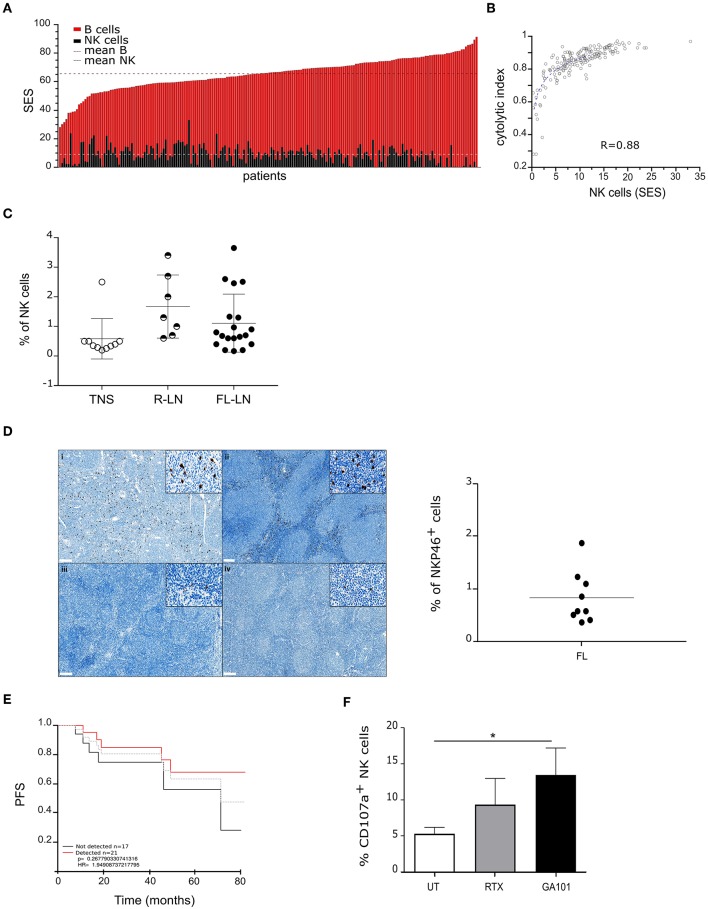
Functional NK cells infiltrate follicular lymphoma lymh nodes. **(A)** Sample enrichment scores (SES) of NK and B cells in 169 FL patients. **(B)** Correlation of “NK cells” SES with expression levels of the cytolytic index. **(C)** Percent of NK cells in normal tonsil (TNS, *n* = 10), reactive (R-LN, *n* = 7) or FL lymph nodes (FL-LN, *n* = 19). NK cells were discriminated by CD56^+^/CD3^−^ staining by flow cytometry. **(D)** Left panel, Representative sections from FL lymph node with a strong (i, ii) and weak (iii, iv) NKP46^+^ staining (brown). Magnification × 70 (insert, × 800). Right panel, Quantification of NK cells (%) in FFPE FL lymph nodes (*n* = 9). **(E)** Kaplan–Meier estimation of PFS of 169 FL patients according to NK cells detection in biopsies. The gray line represents the mean. **(F)** Autologous degranulation of NK cells in FL samples after treatment or not by RTX or GA101 at 10 μl/ml during 4 h. Histograms represent the mean ± sem of 6 independent FL samples. ^*^p < 0.05 compared to untreated condition.

We first analyzed a large cohort (*n* = 169) of public available transcriptome (29–34) datasets of FL biopsies by data mining. The composition of tumor infiltrating leucocytes was deconvolved as depicted (35) and SES of B and NK cells, corresponding to a transcriptomic signature of 79 genes, respectively (Supplementary Figure 1) was determined. As shown in Figure 1A and in line with the FL cell of origin, “B cell” SES is predominant in all samples (mean SES for “B cells”: 65.4; range: 28.1–91.3). Interestingly, we find that “NK cell” SES, with a mean of 9.05 (range: 0.01–33.03), correlates with a cytolytic index (Figure 1B).

Next, we measured NK cell percent by flow cytometry on a series of fresh human FL biopsies, normal tonsil and reactive lymph nodes (see Supplementary Figure 2, Supplementary Tables 1, 2 for sample characteristics), and show that number of infiltrated NK cells increases in both FL (mean: 1.11, range: 0.2–3.35) and reactive lymph nodes (mean: 1.67, range: 0.6–3.4) compared to normal tonsil (mean: 0.584, range: 0.2–2.5) (Figure 1C).

Immunohistochemistry of NKP46 on FFPE FL lymph nodes reveals that NK cells mainly localize at the interfollicular spaces (Figure 1D, left panel). However, we observe a heterogeneity among FL patients with patients presenting a strong NKP46 labeling (Figure 1Di, Figure 1Dii) and others presenting a weak labeling (Figure 1Diii, Figure 1Div). Quantification of NK cell percent on this FL cohort validates results obtained by flow cytometry (Figure 1D, right panel).

We wondered whether the presence of NK in FL biopsies could be correlated with patient survival. Thus, we analyzed by data mining (*n* = 169) public available transcriptome datasets of FL biopsies on their content of NK and determined the PFS. Interestingly, patients with detectable NK cells within the biopsy seem to display longer PFS than patients without NK within the tumor (Figure 1E).

Finally, NK functionality was assessed *in situ*. For this, fresh FL biopsies (bulk) were dissociated, treated with anti-CD20 mAbs and CD107a expression was evaluated by flow cytometry. To reveal cytotoxic granule release by lymph node infiltrating NK cells, the following gating strategy was used: live cells were first selected based on their SSC and FSC, then doublet were excluded and finally, on single cells, CD107a expression was evaluated on CD56^+^/CD3^−^ gated cells (Supplementary Figure 3). In contrast to that observed after treatment with anti-Her2 control mAb, both anti-CD20 mAbs induce an increase of cytotoxic granule release, with a more pronounced effect observed after GA101 treatment (Figure 1F).

Our results show that cytotoxic NK cells infiltrate FL lymph nodes and are responsive to GA101- or RTX-induced ADCC.

### NK Cells Mediate Anti-CD20 mAbs-Induced FL Cell Death in 3D Co-culture

Since FL tumors are organized in masses enriched with FL cells and other cell types such as NK cells (37), we asked whether FL cells aggregates grown as MALC (15, 19, 24, 25) could be sensitive to NK mediated ADCC. Accordingly, MALC were co-incubated *in vitro* with allogeneic NK cells isolated from healthy individual PBMC in the presence or not of RTX or GA101. TTZ was used as control in some key experiments.

**Figure 2:**
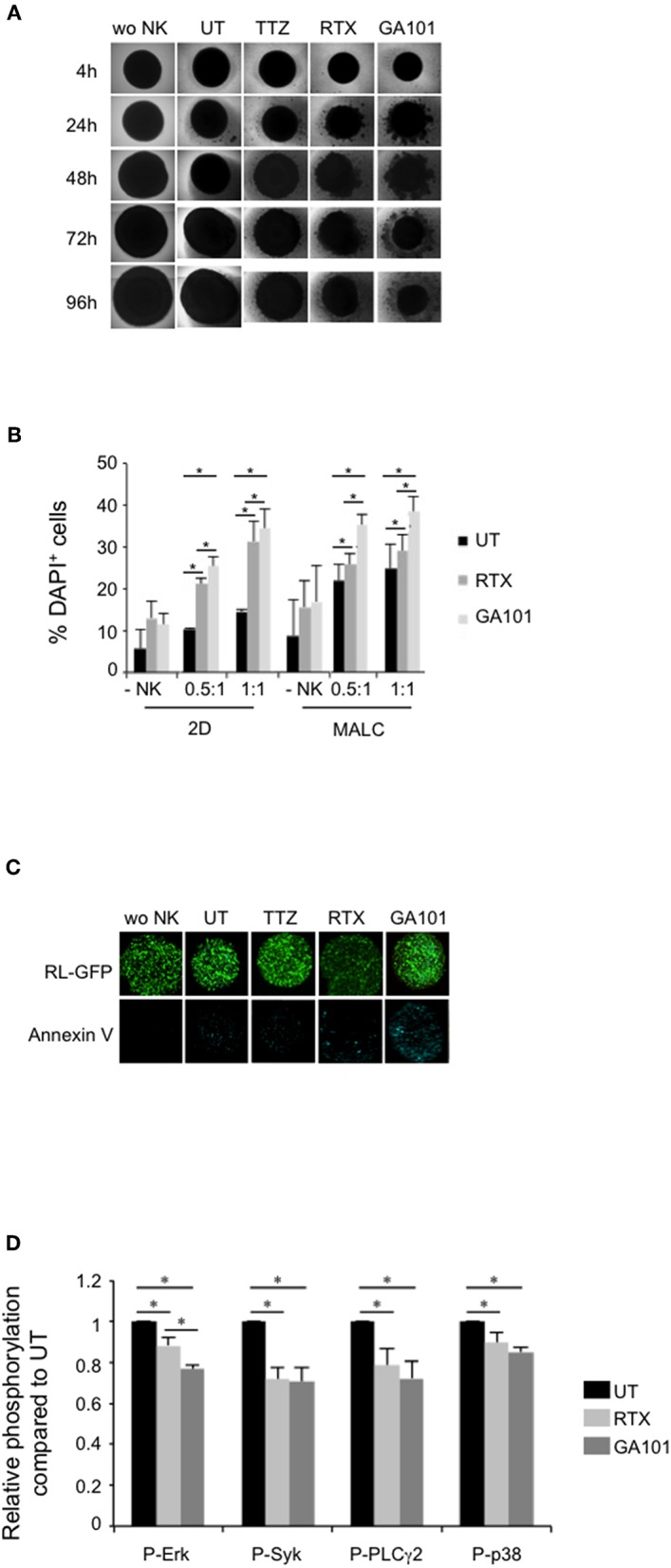
MALC targeting by anti-CD20-induced activation of NK cells. **(A)** Visualization of MALC structure. MALC of RL cells at day 10 of culture were incubated or not (wo NK) with NK cells at ratio E:T 0.5:1 and treated or not (UT) by TTZ, RTX or GA101 at 10 μl/ml. MALC structure was observed after 4 to 96 h with an inverted Nikon Eclipse TE200 microscope at magnification ×40. Pictures are representative of five independent experiments. **(B)** Determination of target cell death by flow cytometry. MALC of RL cells at day 10 of culture were incubated or not (-NK) with NK cells at two different ratios E:T 0.5:1 and 1:1 and treated or not (UT) with mAbs at 10 μl/ml. After 4 h, target cell death was determined by flow cytometry using DAPI staining on CD19^+^ gated cells. Results are mean ± sem of five independent experiments. ^*^p < 0.05 compared to UT condition or between anti-CD20 mAbs. **(C)** Visualization of target cell death. MALC of RL-GFP cells at day 10 of culture were incubated or not (wo NK) with NK cells at ratio E:T 10:1 and treated or not by TTZ, RTX or GA101 at 10 μl/ml. After 4 h, MALC cell death was visualized by Annexin V staining and observed with a confocal microscope. **(D)** Determination of phosphoflow in target cells. MALC of RL cells at day 10 of culture were co-cultured with NK cells at ratio E:T 0.5:1 and treated or not (UT) by RTX or GA101 at 10 μl/ml. After 4 h, signaling in CD19^+^ cells was investigated by flow cytometry. Results are mean ± sem of five independent experiments. ^*^p < 0.05 compared to UT condition or between anti-CD20 mAbs.

Co-culture global architecture was analyzed by bright field microscopy. We evidence that NK cells are able to reduce MALC's area in absence of treatment (Figure 2A, “UT”). Upon anti-CD20 mAbs treatment, we observe a progressive destructuration of the MALC starting as soon as 4 h post-treatment, compared to the negative control anti-Her2 mAb treatment (TTZ). GA101 seems to impact more strongly MALC structure than RTX (Figure 2A).

Cell death of FL cultured in suspension (2D) or in aggregates (3D) in presence or not of NK cells was measured by evaluating DAPI staining by flow cytometry after 4 h of contact (Figure 2B). We confirm that NK cells, in absence of anti-CD20 mAbs treatment, are able to induce FL cell death. This is more pronounced when FL cell are cultured in 3D (MALC) compared to FL cells cultured in suspension (2D) (Figure 2B, “UT”). Anti-CD20 mAbs tend to increase DAPI^+^ cells when cultured without NK cells, supporting the induction of direct effect (15). This phenomenon is significantly enhanced in presence of NK cells, whatever the ratio used, supporting the induction of ADCC (Figure 2B).

To visualize anti-CD20 mAbs-induced apoptosis, we performed annexin V labeling of MALC developed with RL-GFP cells and co-cultured with NK cells. After 4 h, apoptotic cell death is clearly enhanced by GA101 as observed by fluorescent microscopy (Figure 2C). These results suggest that the above-depicted destructuration of MALC results from ADCC in presence of GA101 and, to a lower extent, RTX.

The viability of FL cells usually relies on activated intracellular signaling pathways (38) that are affected by treatment with RTX and GA101 (15, 39, 40). Such signaling pathways were evaluated in CD19^+^ cells (FL cells) of NK/MALC co-culture treated with anti-CD20 mAbs by performing intracellular immunolabelling of phosphoproteins *via* flow cytometry, named phosphoflow (Figure 2D). We found that the levels of Erk, Syk, PLCγ2, and p38 MAPK phosphorylation in FL cells decrease within 4 h of treatment by RTX or GA101 mAbs. Among the four kinases investigated, ERK is significantly more inhibited by GA101 compared to RTX. These results indicate that ADCC conditions reduce survival signaling in MALC.

### Cytolytic and Cytokine NK Cell Responses in ADCC of MALC

**Figure 3:**
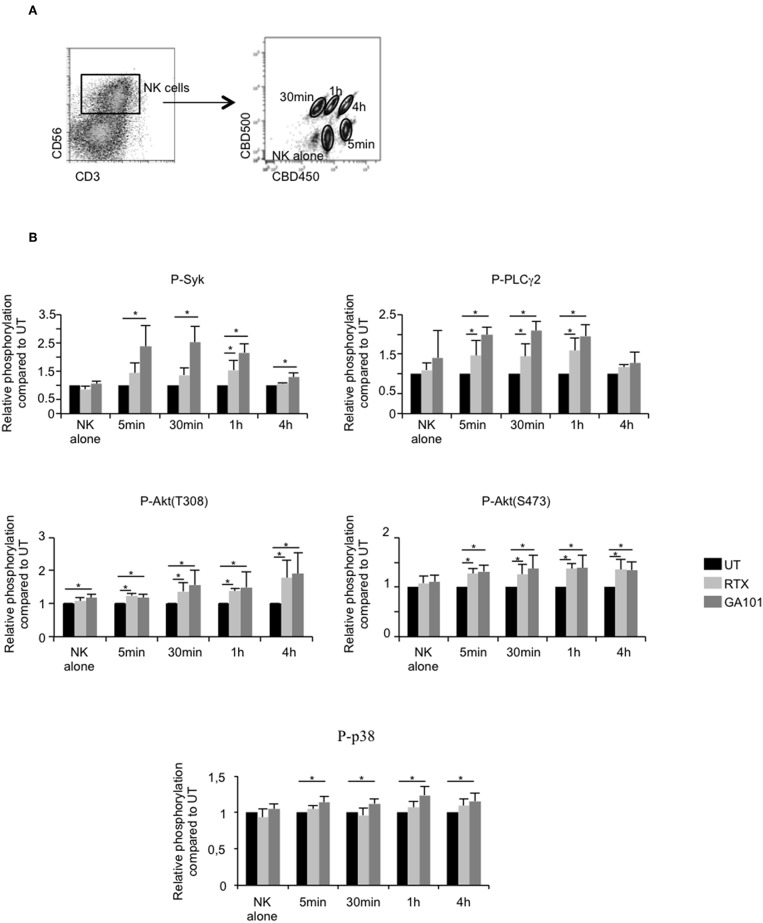
Downstream FcγRIIIa signaling in activated NK cells. MALC of RL cells at day 10 of culture were incubated or not with NK cells at ratio E:T 0.5:1 and treated or not (UT) with mAbs at 10 μl/ml. Five min, 30 min, 1 h, or 4 h after treatment, cells were barcoded individually with different concentrations of CBD dyes as described in methods and intracellular phosphoflow was analyzed. **(A)** Representative cytogram. Example of 5 different samples deconvoluted after fluorescent barcoding with different concentrations of CBD450 and CBD500. **(B)** FcγRIIIA intracellular signaling. Phosphorylation status of Syk, PLCγ2, AKT, and p38 was determined by flow cytometry on NK cells. Histograms represent the ratio of MFI of treated compared to UT conditions. Results are mean ± sem of five independent experiments. ^*^p < 0.05.

The above results suggest that MALC destruction is due to NK cell-mediated ADCC reaction. Therefore, the signaling cascade triggered by FcγRIIIA activation was analyzed at successive time points after anti-CD20 mAbs treatment by deconvolution of intracellular phosphoprotein immunostaining of NK cells previously bar-encoded with CBD 500/CBC450 and flow cytometry (Figure 3A). We analyzed the relative phosphorylation of Syk, PLCγ2, Akt (T308 and S473), and p38 MAPK in NK cells co-cultured or not with MALC in the presence or not with anti-CD20 mAbs. Treatment of NK cells with RTX or GA101 do not affect FcγRIIIA downstream signaling (Figure 3B, “NK alone”). However, once co-cultured with MALC, NK cells exhibit activation of Syk, PLCγ2, and Akt as soon as five min after exposure to anti-CD20 mAbs. The phosphorylation of p38 MAPK appears weaker and most consistently seen after 1 h of exposure to MALC with GA101 (Figure 3B). These experiments demonstrate that anti-CD20 mAbs engage FcγRIIIA downstream signaling cascade in NK cells only when cultured with MALC.

**Figure 4:**
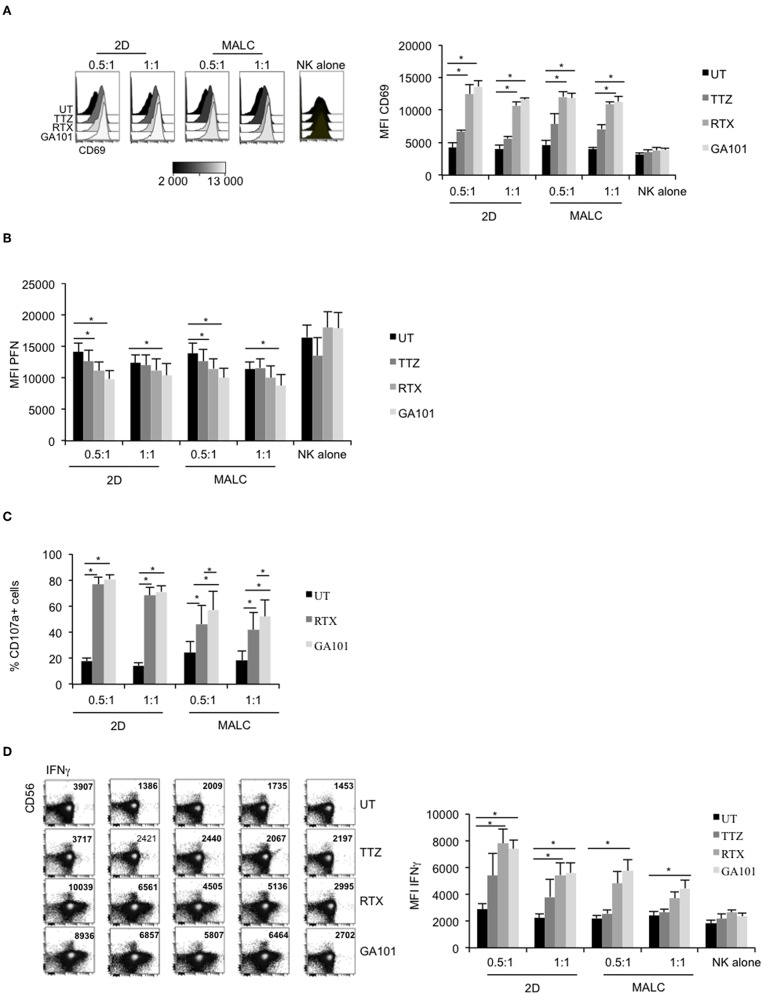
Degranulation and cytokine release of activated NK cells. RL cells in suspension (2D) or MALC of RL cells at day 10 of culture were incubated or not with NK cells at ratio E:T 0.5:1 or 1:1 and treated or not (UT) with mAbs at 10 μl/ml. After 4 h, CD69 **(A)**, PFN **(B)**, CD107a **(C)**, and IFNγ **(D)** expression were detected by flow cytometry by gating on CD56^+^/CD3^−^ NK cells. Results are mean ± sem of five independent experiments. ^*^p < 0.05 compared to UT condition or between anti-CD20 mAbs.

NK cell activation, characterized by up-regulated cell surface expression of CD69, was tested on NK cells exposed to MALC opsonized with the anti-CD20 mAbs. TTZ, used as negative control, does not significantly affect CD69 expression level (Figure 4A). Interestingly, without target cells, anti-CD20 mAbs do not activate NK cells (Figure 4A, “NK alone”), confirming the above results (Figure 3B, “NK alone”). However, CD69 amount is increased when NK are in presence of both target cells (2D and MALC) and anti-CD20 mAbs (either RTX or GA101) (Figure 4A). During ADCC, activated NK cells release their lytic granules containing PFN and granzymes through their lytic immunological synapse with targets. We thus monitored by flow cytometry NK cell degranulation by immunomonitoring intracellular PFN (Figure 4B) and cell surface CD107a expression (Figure 4C). NK cells alone, even in presence of anti-CD20 mAbs, do not degranulate (Figure 4B, ‘NK alone'). By contrast, the decrease of their intracellular PFN is evident upon exposure to FL cells treated with either anti-CD20 mAb, but not upon exposure to FL cells treated with control mAb (TTZ). The NK cell degranulation is observed with FL cell cultured in suspension or in MALC, although GA101 seems to have a better efficacy than RTX in both settings (Figures 4B,C). NK cells synthesize and release INFγ upon activation, so we tested by flow cytometry whether anti-CD20 mAbs could induce such cytokine response when NK cells were co-cultured with FL cells. Whatever the type of cell culture (suspension vs. 3D), RTX and GA101 induce IFNγ production (Figure 4D). However, and as shown for NK degranulation, GA101 is more potent than RTX when NK cells are cultured with MALC compared to FL cells cultured in suspension. No effect of anti-CD20 mAbs on IFNγ production is observed when NK cells are cultured without target cells. Altogether, these results show that anti-CD20 mAbs are able to activate NK cells only when cultured with FL cells (suspension or 3D), with an enhanced efficacy of GA101 compared to RTX.

### NK Cells Mediate Anti-CD20 mAb-Induced ADCC of FL Xenografts

**Figure 5:**
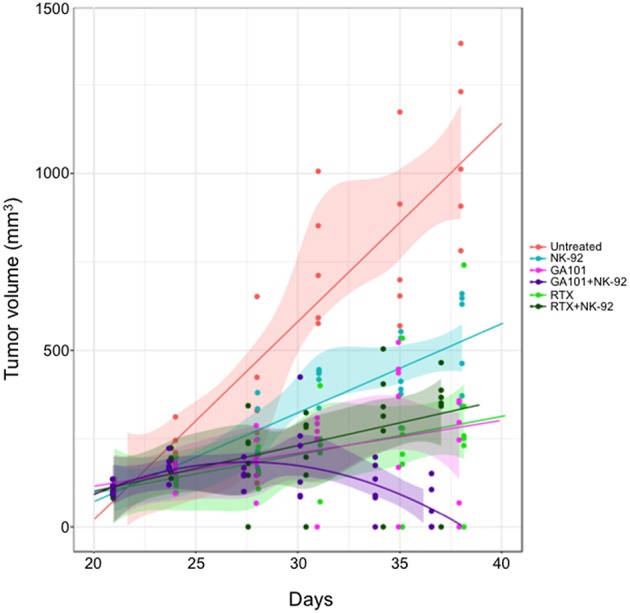
Anti-CD20 mAbs induced NK-mediated ADCC *in vivo*. SCID-Beige mice were engrafted with RL cells. Six cohorts of six animals were treated or not (red) with RTX (30 mg/kg, green), GA101 (30 mg/kg, pink) in the presence or not of NK-92 cells (dark green and violet, respectively). Over time, tumor volume (TV) was measured with a caliper. Regression curves representing the mean of TV in mm^3^ were plotted (lines).

The above results validate the 3D FL co-culture with NK cells as a relevant model for anti-CD20 MAbs-induced ADCC of FL *in vitro*. Whether this reaction operates likewise *in vivo* remains unclear. Thus, we sought to validate NK cell-mediated ADCC of FL cells *in vivo*, by engrafting human FL cells in SCID-beige mice that were further treated with human NK cells and a single dose of RTX or GA101. The volume of FL tumors was then compared at various time points in mice receiving different regimens. We found that NK-92 cells alone or anti-CD20 mAbs alone slow down tumor growth with a stronger effect in presence of GA101 compared to RTX. The presence of NK cells in the regimen does not influence the effect mediated by RTX as attested by a non-significant *p*-value (Supplementary Figure 4). However, when associated with GA101, a strong reduction of TV is observed, supporting ADCC induced by the glycoengineered anti-CD20 mAb *in vivo* (Figure 5).

## Discussion

A major challenge in cancer research is to integrate the complexity of the tumor microenvironment (TME) and the necessity to take into account the host immunological setting. An innovative way to address these problems is to exploit the benefit yielded by 3D co-culture models that tend to recapitulate the tumor architecture and ecosystem. Except the MALC model we developed, which “mimics” the pathology by its architectural organization, gene profiling, response to treatment and immune escape profiles (15, 19, 24, 25), no 3D culture model exists for *in vitro* FL studies. Nevertheless, the use of such models is crucial as it is well-established that direct or immune-mediated response to treatment differs according to culture conditions (2D vs. 3D) (15, 19, 24). Indeed, FL cells cultured in 3D vs. 2D suspension are differentially sensitive to anti-CD20 mAbs (15) and chemotherapy (25), strongly supporting the fact that compaction/aggregation induces modifications in the activation status of key enzymes yielding to a stronger efficacy of treatment by inhibiting their overactivated targets (15, 38). Moreover, we previously reported that FL cell growth in MALC confers innate resistance to cytolytic lymphocytes (24). However, this model is “minimalist” as it is composed by one cellular type. Thus, strong efforts are needed to increase its complexity by incorporating cells from TME rendering it more relevant to the pathology. Here and combined with our recent publication (19), we present strong evidences that 3D co-cultures of FL cells with NK cells or Tγδ represent a new pertinent, robust, easily handled model allowing, not only ADCC determination, but also evaluation of drug combination efficacy, drug penetration and immune cell distribution. We are convinced that this co-culture model should be used for *in vitro* studies in place of 2D cultures as it takes into account parameters missing in suspension cultures such as tumor spatial organization, distribution of immune cells, penetration and gradient of drug, modification of gene expression, increase of survival signaling. This co-culture model developed with FL cell lines is robust, low-cost and reproducible. It allows to address the question of mechanism of action of drugs, therapeutic combination efficacy and to identify mechanisms of resistance. We concede that this model is still far away from physiopathology and the development of patient-derived lymphoma spheroids (PDLS) (from FL biopsies or blood) is crucial. PDLS are expected to better understand FL pathology but also, in precision medicine, to improve drug development. The evolution of FL research toward personalized medicine by using such 3D co-culture models integrating TME instead of murine models which are expensive, time and resource consuming, complex to set up, and not always realists of the physiopathology is a new challenge.

Here, we determined signaling cascade downstream FcγRIIIa after anti-CD20 mAbs treatment and observe an activation of Syk, PLCγ2, AKT, and p38 reflecting activation of NK cell as attested by cytokine, PFN and granzyme B release only when immune cells were cultured in presence of target cells. The fact that GA101 tends to activate more efficiently than RTX downstream FcγRIIIA signaling was expected, as this humanized type II anti-CD20 mAb has been glycoengineered to exhibit a stronger affinity to CD16 and consequently increase ADCC (9). However, unlike monocellular 3D culture, when co-cultured with NK cells, FL cells are as sensitive as cells cultured in suspension. This suggests that NK and anti-CD20 mAbs are able to penetrate the MALC to kill tumor cells as it has been described for γδ T cells (19). On target cells, GA101 seems to induce a stronger inhibition of over-activated kinases leading to a more potent cell death than those induced by RTX. This was also expected as GA101 is a more potent inducer of direct cell death than RTX *in vitro* (in 2D or 3D models) (15) and *in vivo* (16).

NK cells are important components of the innate immune response against malignant cells. In FL, NK cell amount in peripheral blood is associated with clinical outcomes as low cell number correlates with worse overall survival and shorter time to progression (22, 23). This seems to be correlated with a decrease of RTX mediated ADCC in FL patients (41). Here, we show that cytolytic NK are present in FL biopsies and are functional. When incubated with anti-CD20 mAbs, NK are able to kill tumoral cells thus confirming their role in ADCC *in vivo*. Nevertheless, mAbs alone are not as efficient as expected in patients and combination with other drugs (immune check point [ICP] blockers, microenvironment targeting, kinases inhibitors…) need to be tested to improve patient management. In this context, and based on our recent findings showing that PD-1 hampers γδ T cells-mediated ADCC (19), it could be interesting to evaluate the expression of this ICP on NK and its blockade on their anti-tumoral properties against 3D FL cultures in combination to anti-CD20 mAbs for example. This is important to be considered as research in this field has been relatively neglected and only few very recent studies explored the role of PD-1 on NK cells in solid cancers (42, 43) and in DLBCL (44). Thus, further investigations are needed to explore the therapeutic potential of this attractive subtype of immune cells mediating non-MHC-restricted killing of tumor cells (45).

## Data Availability

The raw data supporting the conclusions of this manuscript will be made available by the authors, without undue reservation, to any qualified researcher.

## Ethics Statement

Tissue samples were collected and processed following the standard ethical procedures of the Helsinki protocol, after obtaining written, informed consent from each donor and local ethical committee approval for the study (Comité de Protection des Personnes Sud-Ouest et Outremer II).

## Author Contributions

ED, CR, and CB designed the experimental strategy, organized the experiments, and collected and analyzed the data. PG and JB performed cell isolation and flow cytometry analysis on FL samples. ED and CR isolated and cultured NK cells from healthy donors. ED, CR, and JB performed *in vitro* experiments. AT, EP, and CD performed and analyzed *in vivo* experiments. MT generated SES software and performed analysis by data mining with CR, CB, J-JF, and CL. CL and PG selected FL biopsies and performed and analyzed IHC experiments. MP, AS, and CK provided the experimental advices and helped for manuscript discussion. CB wrote the manuscript.

### Conflict of Interest Statement

At the time of their implication in the study, ED and AS were employees of Institut Roche. CK is an employee of Roche and holds Roche stock and patents. The remaining authors declare that the research was conducted in the absence of any commercial or financial relationships that could be construed as a potential conflict of interest.

## Abbreviations

ADCC: Antibody Dependent Cell Cytotoxicity
FL: Follicular Lymphoma
ICP: Immune Check Point
IFN: Interferon
mAb: monoclonal Antibody
MALC: Multicellular Aggregates of Lymphoma Cells
NHL: Non-Hodgkin's Lymphoma
NK: Natural Killer
PDLS: Patient Derived Lymphoma Spheroids
PFN: Perforin
PFS: Patient Free Survival
RTX: Rituximab
SES: Sample Enrichment Score
TME: Tumor Microenvironnement
TTZ: Trastuzumab
TV: Tumor Volume.
