# Supplementary figures and images for: A Tridimensional Model for NK Cell-Mediated ADCC of Follicular Lymphoma
Source: Front Immunol. 2019 Aug 14;10:1943. doi: 10.3389/fimmu.2019.01943 (PMC6702952; doi:10.3389/fimmu.2019.01943)

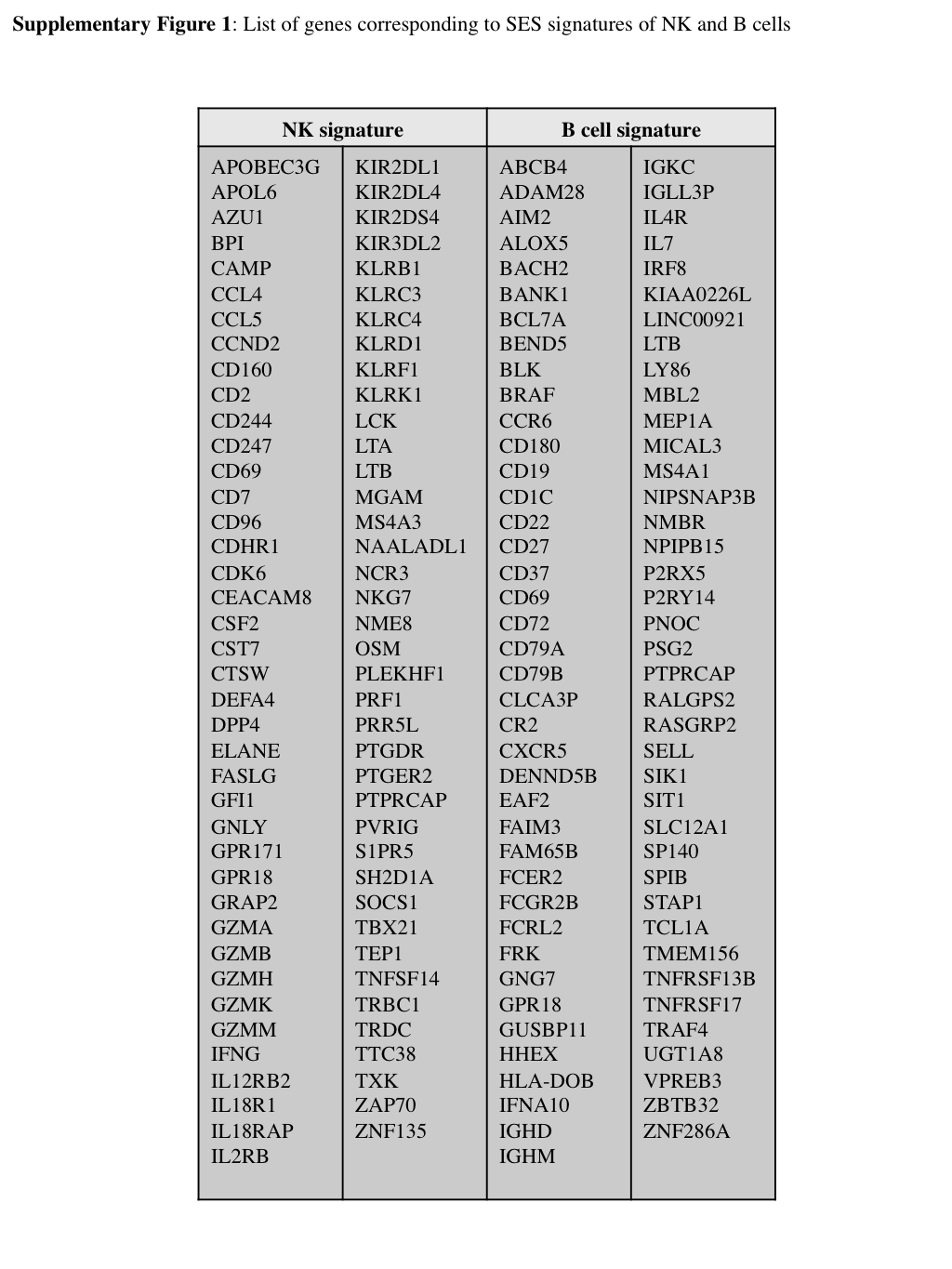

Supplement: Supplementary file 1 [file Image_1.TIFF]

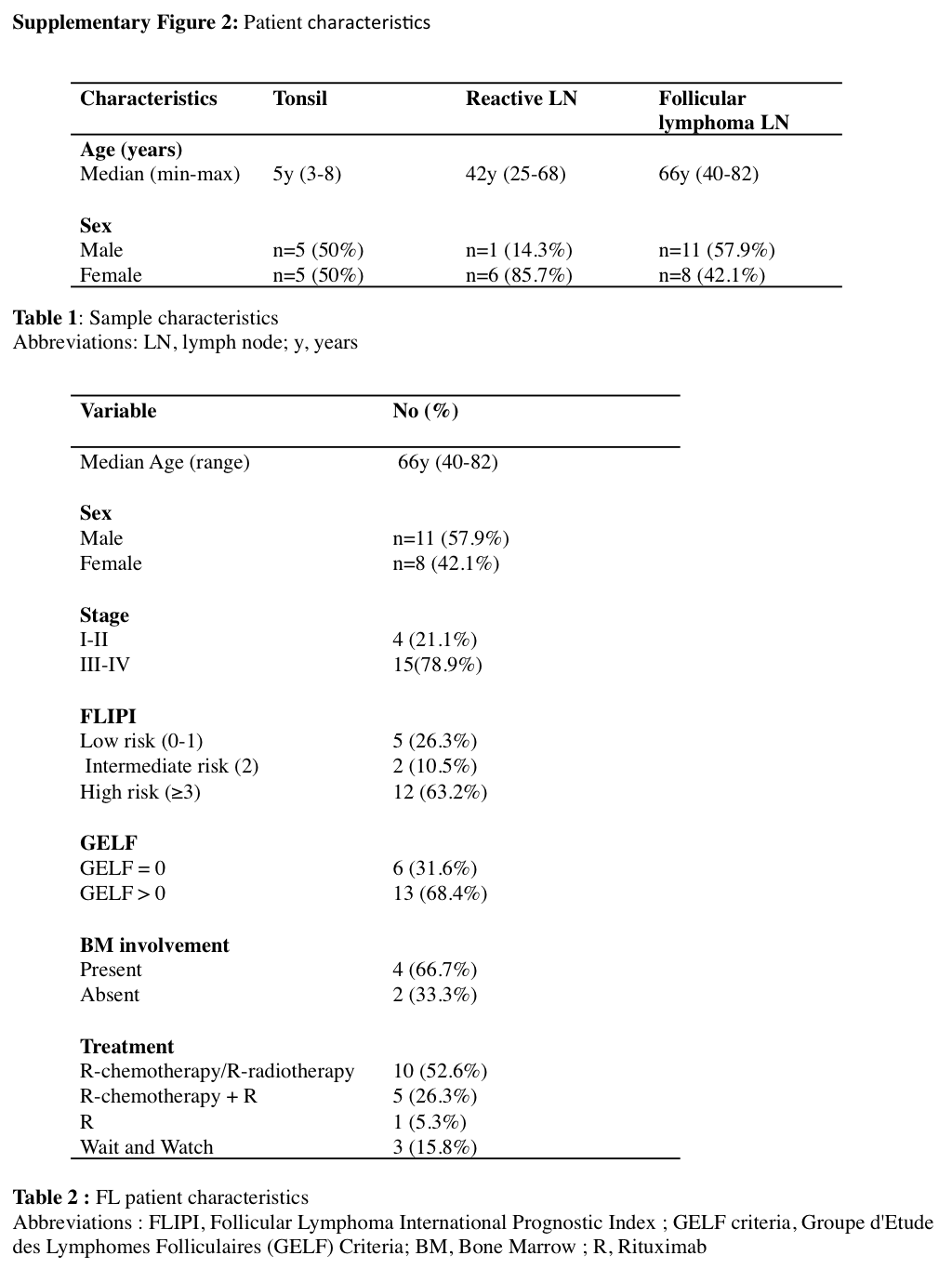

Supplement: Supplementary file 2 [file Image_2.TIFF]

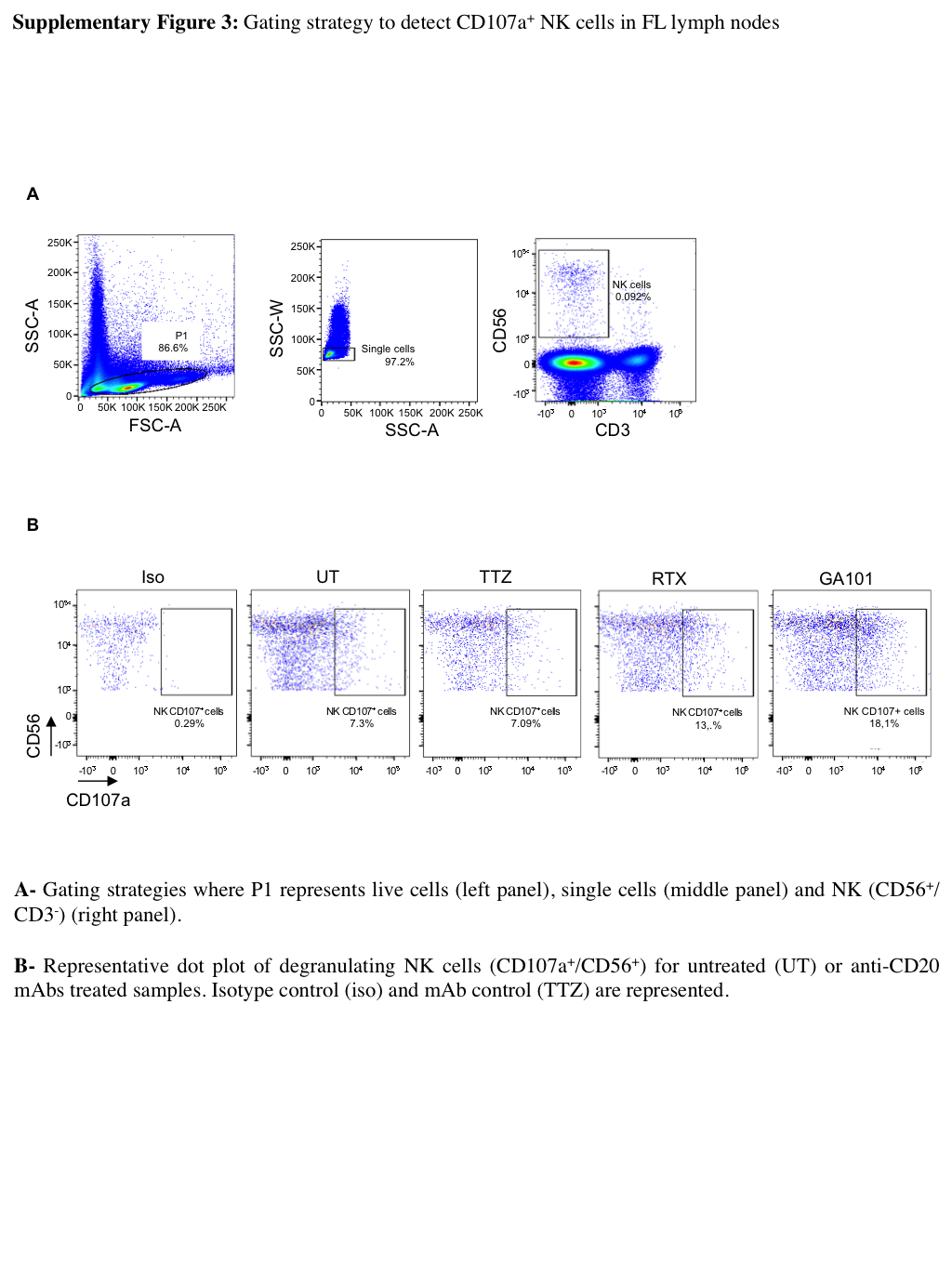

Supplement: Supplementary file 3 [file Image_3.TIFF]

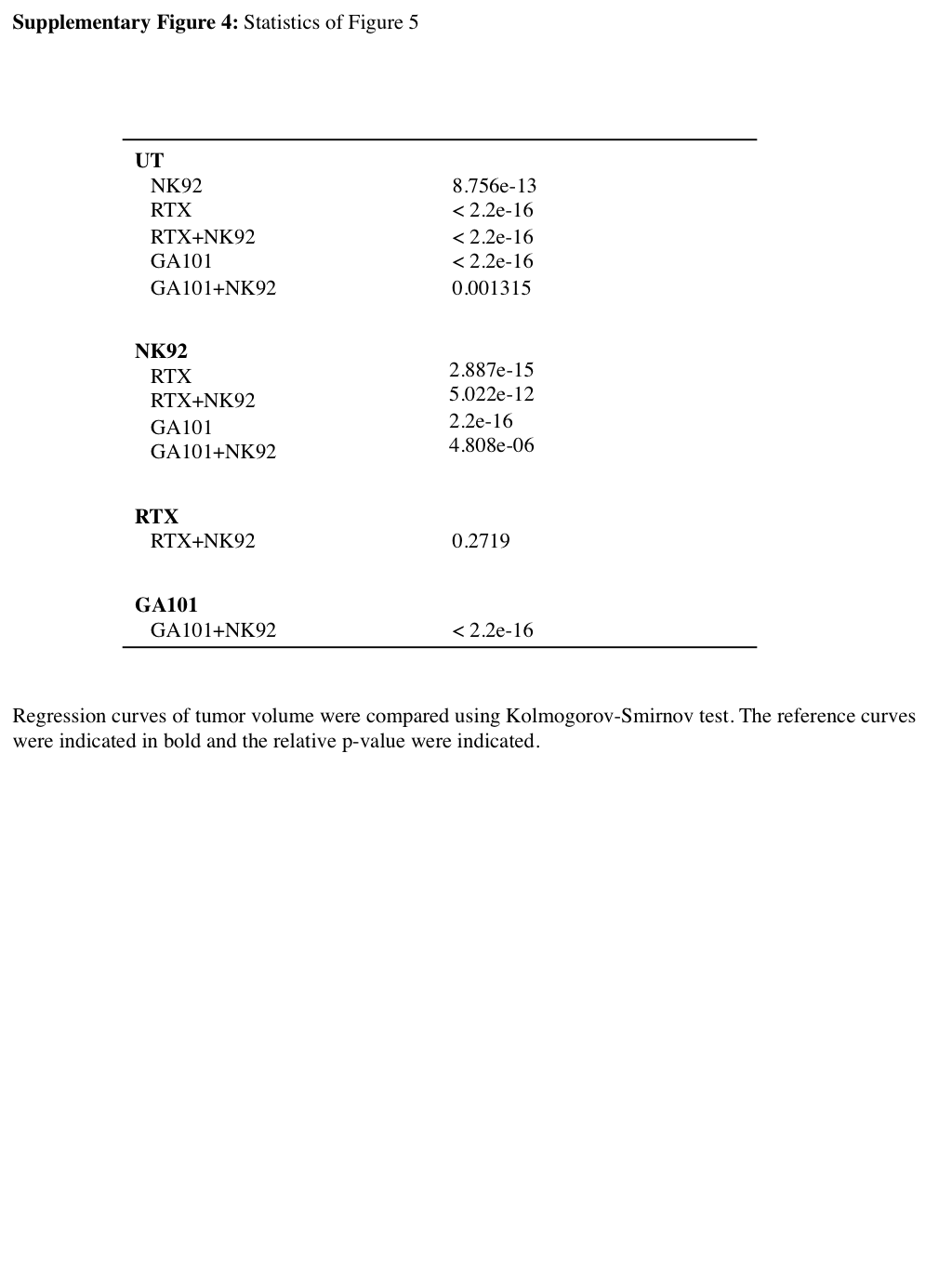

Supplement: Supplementary file 4 [file Image_4.TIFF]
